# Authors’ Reply: Urological Content Accuracy as an Unmeasured Dimension in Generative AI Chatbot Communication About Sexual Health After Prostate Cancer

**DOI:** 10.2196/109004

**Published:** 2026-09-15

**Authors:** Mats Christiansen, Henrik Eriksson, Lisbeth Fagerström

**Affiliations:** 1 Department of Public Health and Caring Sciences, Uppsala University, BMC, Husargatan 3, Uppsala, 752 37, Sweden, 46 18-471 66 20; 2 Department of Natural and Health Sciences, Faculty of Science and Engineering, Åbo Akademi University, Vaasa, Ostrobothnia, Finland; 3 Section for Health Promotion and Care Sciences, University West, Trollhättan, Västra Götaland, Sweden

**Keywords:** auto-netnography, netnography, prostate cancer, sexual minority men, generative AI chatbots

We sincerely thank the authors [1] for their thoughtful engagement with our article [2] and for raising important questions regarding the clinical accuracy of AI-generated health information. We welcome this opportunity for scientific dialogue.

Clinical accuracy is unquestionably an essential dimension of generative AI in health care. The letter and our original article illuminate different dimensions of the same phenomenon. The letter examines the clinical validity and completeness of AI-generated information, whereas our study examined how that information is framed, enacted, and experienced within a situated interaction concerning sexual health after prostate cancer. These perspectives are complementary because they address different dimensions of AI-mediated supportive care.

This distinction was fundamental to the design of our study. As stated in the original article [2], the study was “not designed to evaluate clinical effectiveness, safety, or generalizable performance of chatbots,” but instead sought to examine “how responses are framed and experienced in sexual minority contexts.” Consistent with contemporary netnographic scholarship, our analytical interest, therefore, lies in the interaction itself rather than in validating the medical correctness of individual chatbot responses [3-5].

The observations presented in the letter [1] illustrate this distinction. The authors note, for example, that none of the chatbots challenged the hypothetical assumption that both radical prostatectomy and radiotherapy would likely include hormonal therapy. We agree that this represents an important clinical observation and precisely the type of finding that a benchmarking study is designed to identify. Within our study [2], however, the hypothetical patient scenario functioned as a standardized interactional prompt rather than a test of guideline concordance. It also highlights an important feature of real-world human-AI interaction: people seeking health information may formulate prompts from incomplete or inaccurate understandings of their condition or treatment. Whether generative AI identifies, challenges, or reproduces such assumptions represents an important question for future research.

Similarly, the observations concerning contextual inaccuracies reflect these complementary perspectives. Our study reported such observations descriptively because they formed part of the interactional experience under investigation rather than constituting outcome measures of clinical reliability.

We, therefore, do not view the letter as challenging the central contribution of our study. Rather, it complements that contribution by examining another important dimension of AI-mediated supportive care. Clinical benchmarking establishes whether AI-generated information is medically reliable. Interpretive inquiry examines how that information becomes meaningful through interaction. Together, these perspectives provide a more comprehensive understanding of generative AI as a digital adjunct in supportive cancer care.
